# Nanofiber Alignment Regulates NIH3T3 Cell Orientation and Cytoskeletal Gene Expression on Electrospun PCL+Gelatin Nanofibers

**DOI:** 10.1371/journal.pone.0154806

**Published:** 2016-05-19

**Authors:** Timothy Fee, Swetha Surianarayanan, Crawford Downs, Yong Zhou, Joel Berry

**Affiliations:** 1 Department of Biomedical Engineering, UAB, Birmingham, Alabama, United States of America; 2 Department of Ophthalmology, Center for Ocular Biomechanics and Biotransport, UAB, Birmingham, Alabama, United States of America; 3 Department of Medicine, Division of Pulmonary, Allergy and Critical Care Medicine, UAB, Birmingham, Alabama, United States of America; Osaka University, JAPAN

## Abstract

To examine the influence of substrate topology on the behavior of fibroblasts, tissue engineering scaffolds were electrospun from polycaprolactone (PCL) and a blend of PCL and gelatin (PCL+Gel) to produce matrices with both random and aligned nanofibrous orientations. The addition of gelatin to the scaffold was shown to increase the hydrophilicity of the PCL matrix and to increase the proliferation of NIH3T3 cells compared to scaffolds of PCL alone. The orientation of nanofibers within the matrix did not have an effect on the proliferation of adherent cells, but cells on aligned substrates were shown to elongate and align parallel to the direction of substrate fiber alignment. A microarray of cyotoskeleton regulators was probed to examine differences in gene expression between cells grown on an aligned and randomly oriented substrates. It was found that transcriptional expression of eight genes was statistically different between the two conditions, with all of them being upregulated in the aligned condition. The proteins encoded by these genes are linked to production and polymerization of actin microfilaments, as well as focal adhesion assembly. Taken together, the data indicates NIH3T3 fibroblasts on aligned substrates align themselves parallel with their substrate and increase production of actin and focal adhesion related genes.

## Introduction

Using electrospun materials as scaffolds for engineering tissue replacements remains a promising research area. Electrospun scaffolds can be fabricated from numerous biodegradable materials and their nanofibrous structure can possess features which mimic the architecture of the native extracellular matrix (ECM) of many tissues. The electrospinning apparatus only requires a few components and is highly customizable to produce tailored nanofibrous matrices. Electrospun scaffolds composed of highly aligned fibers are of particular interest due to their ability to modulate many cellular behaviors. Cells cultured on substrates with an oriented microtopology have been shown to behave differently than cells on randomly oriented or smooth materials [1]. Recent examples of substrate topology regulated cell behaviors include: inducing alignment in nerve regeneration, influencing proliferation of cardiac myocytes, and modulating myofibroblast differentiation [2–6].

Many materials have been successfully electrospun into nano-scale fibers. Polycaprolactone (PCL) is frequently used for electrospinning as it possesses several desirable mechanical properties. Specifically, PCL has a high elastic modulus and is simultaneously a hyper-elastic material and can be deformed to over 100% strain prior to failure, though some sources report a lower strain at failure [7]. Additionally, PCL is a biocompatible material with no reported cytotoxicity and is degradable on a timescale of months to years. These properties make PCL an attractive option for electrospun scaffolds, but scaffolds produced from 100% PCL fibers are limited by poor cell adhesion due to the relatively high hydrophobicity of PCL. To improve the hydrophilicity of PCL scaffolds, researchers have: coated PCL scaffolds with a bioactive molecule such as collagen or fibronectin, or incorporated other materials in to the PCL fibers [8–10]. At first, collagen was widely incorporated into the electrospinning solution with PCL, however as questions arose about the secondary structure of collagen post-electrospinning, researchers began replacing collagen with gelatin in electrospinning solutions [11], [12].

Prior studies of substrate induced gene expression identify the cellular substrate as an important regulator of cellular behavior. Substrate topology induced changes in cell morphology have been documented in a litany of cell types including: neural progenitor cells, mesenchymal stem cells, smooth muscle cells, and Schwann cells [13–15]. Furthermore, various studies have shown substrate topology to be a regulator of gene expression in adherent cells. Neural progenitor cells have been shown to express neural differentiation markers when grown on aligned nanofibers over random fibers [13], [16]. Additionally, pre-osteoblasts show an increase in bone specific markers on aligned fibers over random fibers [17]. However, there is relatively little literature on the influence of substrate topology on fibroblasts. One study, using NIH3T3 fibroblasts, found that fiber orientation was a strong influence of cell morphology and speculated that this would also induce a change in gene expression [18].

In order to examine the role of the alignment of a nanofibrous substrate on the gene expression profile of adherent fibroblasts, we have fabricated and characterized an electrospun matrix of nanofibrous PCL+Gel fibers possessing a controlled orientation. We have further quantified the physical properties of the substrate as well as the response of the adherent cells in terms of growth and expression of cytoskeleton regulation genes.

## Materials and Methods

### Electrospinning

Electrospinning was performed using an optimized protocol described previously [19]. The electrospinning solution for 100% PCL fibers was formed from a 10% (w/v) solution of PCL (MW: 70 kDa—90 kDa, Sigma, St. Louis, MO) in a 50:50 mixture of dichloromethane and dimethylformamide (Fisher Scientific, Pittsburg, PA). Electrospinning solutions used to form PCL+Gel fibers were made from polycaprolactone (PCL, Sigma, St. Louis, MO) and type A gelatin (Sigma) in a 90/10 (w/w) ratio dissolved in Trifluoroethanol (Fisher Scientific, Pittsburg, PA) with 1% acetic acid (Fisher Scientific) to improve miscibility [12]. The PCL+Gel concentration was 10% (w/v) in the electrospinning solution. Either polymer solution was loaded into a 10 mL syringe with a 25 Gauge needle and extruded at a rate of 0.5 mL/hr into a high voltage electric field. The applied static electric potential was +17kV relative to a grounded cylindrical collector 20 cm away. The collector was rotated at high RPMs to collect highly aligned fibers or at very low RPMs to produce randomly oriented fibers. After electrospinning, each sample was put in a vacuum desiccator overnight to remove any residual solvent.

### Polymer characterization

To confirm the presence of gelatin within the scaffold, Fourier Transform Infrared Spectroscopy (FTIR, Nicolet Thermo Scientific, Waltham, MA) operating in ATR mode. To characterize the effect of gelatin on electrospun PCL crystallinity, samples were analyzed using differential scanning calorimetry (DSC, TA Instruments Q series 100 DSC, New Castle, DE), the temperature was swept from -20°C to 80°C at a rate of 10°C/min. The reference enthalpy of fusion value used for 100% crystalline PCL was 135.44 J/g [20]. The hydrophobicity of PCL and PCL+Gel was quantified by measuring the contact angle formed when a drop of water is placed on the surface of the material. After placing a 20 μl drop of water on the surface of a randomly oriented electrospun material, the drop was imaged and the angle was measured using ImageJ (NIH, Bethesda, MD). For each polymer characterization technique, sheets of electrospun material were tested after it was removed from the vacuum desiccator to ensure the removal of any trace of solvent.

### Cell Culture

NIH3T3 cells (American Type Culture Collection, Manassas, VA) were cultured in high glucose Dulbecco's Modified Eagle Medium (Fisher Scientific) supplemented with 10% fetal bovine serum (Fisher Scientific) and 1% penicillin and streptomycin (Fisher Scientific) in a humidified 5% CO_2_ incubator at 37°C.

### Cell Growth Assay

The PicoGreen (Life Technologies, Carlsbad, CA) assay was used to quantify cellular adhesion and proliferation on different substrates according to manufacturer’s instructions. In short, adherent cells on different substrate conditions were rinsed with phosphate buffered saline, then lysed using RIPA buffer at day 0 (4 hours post seeding) and day 4. The lysate was collected and mixed with picogreen reagent and the fluorescence intensity was measured in a plate reader (Biotek, Winooski, VT).

### Imaging

To observe adherent cells on electrospun scaffolds, samples were fixed in 10% neutral buffered formalin, then permeabilized in 0.05% Triton X-100 and stained with Acti-stain 555 phalloidin (Cytoskeleton Inc, Denver, CO) and 4',6-diamidino-2-phenylindole (DAPI). Stained samples were imaged on a Nikon A1 confocal microscope (Melville, NY). Additionally, cells were imaged using electron microscopy. Samples of cell-laden electrospun matrices were prepared for Scanning Electron Microscopy (SEM) by fixing in 10% neutral buffered formalin and chemically dehydrated via serial dilutions in ethanol followed by serial dilutions of hexamethyldisilazane before drying overnight in a vacuum desiccator. After drying, the samples were sputter-coated with gold-palladium and imaged using a Quanta 650 FEG SEM (FEI, Hillsboro, OR) with an accelerating voltage of 10kV.

### Cell Alignment Quantification

To quantify a topology induced cell orientation preference, images of cells acquired via confocal microscopy were processed using ImageJ. The orientations of the cells within an image were determined by using the orientation of the cell nucleus as previously used by other groups [6], [21], [22]. Differences in preferred direction of cells growing on either random or aligned topologies were determined by quantifying percentage of cells within +/- 22.5° of the mean orientation.

### Gene Expression Microarray

Gene expression studies were conducted from cells grown on mats of either random or aligned PCL+Gel fibers covering a 10 cm dish. One day after seeding, the cells were rinsed in PBS to remove non-adherent cells and lysed in TriZol reagent (Life Technologies). Total RNA was isolated using manufacturer’s protocol, briefly, sample was homogenized in TriZol reagent, chloroform was added to remove the protein and DNA components, RNA was purified from the supernatant by isopropanol precipitation and then washed with ethanol before being resuspended in high quality nuclease-free water. The purity and quantity of the isolated RNA was measured by spectroscopy (Biotek). The RNA was reverse transcribed to cDNA using RT^2^ First Strand kit (Qiagen, Valencia, CA), and qPCR was performed using RT^2^ Profiler PCR Array for mouse cytoskeleton regulators (Qiagen). The full list of genes included in the microarray are given in S1 Table.

### Statistical Methods

Statistics were implemented in MATLAB (Mathworks, Natick, MA) using a test appropriate for the comparison being made, specifically t-tests for comparing two groups and ANOVA for multiple groups with a post-hoc Tukey HSD test for multiple comparisons (α = 0.05). Gene expression data from microarray analysis was examined using the Benjamini–Hochberg method [23] to limit the false discovery rate to no more than 2 expected false discoveries out of all the rejected null hypotheses. To increase the likelihood of biological significance, the genes identified as statistically significant were further limited to genes with a fold change larger than 20%. Sample sizes were: N = 4 for contact angle quantification, N = 5 for cell growth assay, and N = 3 for gene expression microarray tests.

## Results

### Production of electrospun fibers

**Fig 1:**
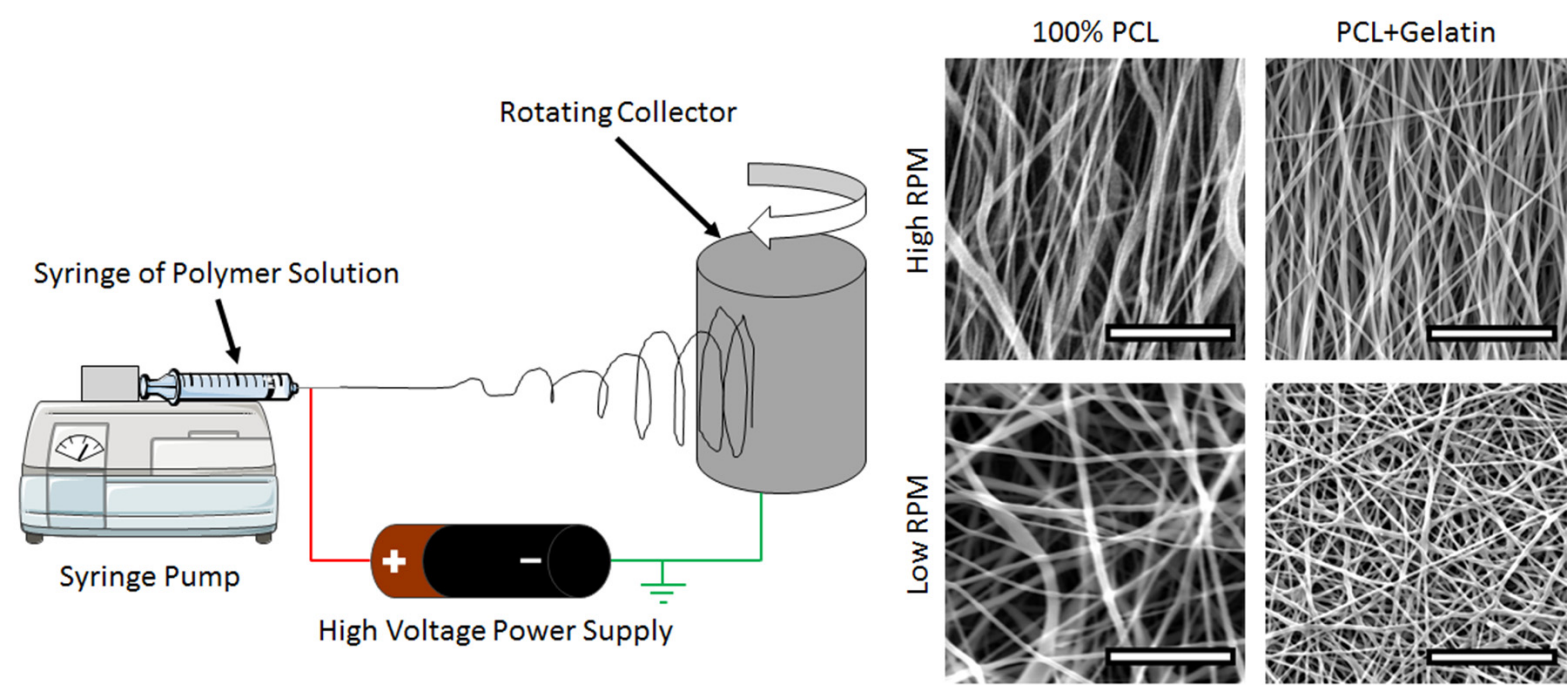
Electrospinning Apparatus and Resulting Nanofibers. A) Diagram of the electrospinning set-up: A polymer solution is extruded from a syringe into a high-voltage electric field towards a grounded cylindrical collector. When the collector is rotating at high RPM, aligned fibers are collected; when the collector is rotating at low RPM, randomly oriented fibers are collected. B) Example SEM images of nanofibers collected under various conditions. Scale bars are 10 microns.

To probe the influence of substrate topology on fibroblast behavior, nanoscale fibrous matrices were produced by electrospinning. Fig 1 illustrates the electrospinning apparatus set-up. Fibers were produced with random or aligned orientations to mimic the different organization of collagen bundles within various tissues. Fig 1 also shows SEM images of 100% PCL fibers and fibers of PCL blended with gelatin (PCL+Gel).

### Characterization of fibers

**Fig 2:**
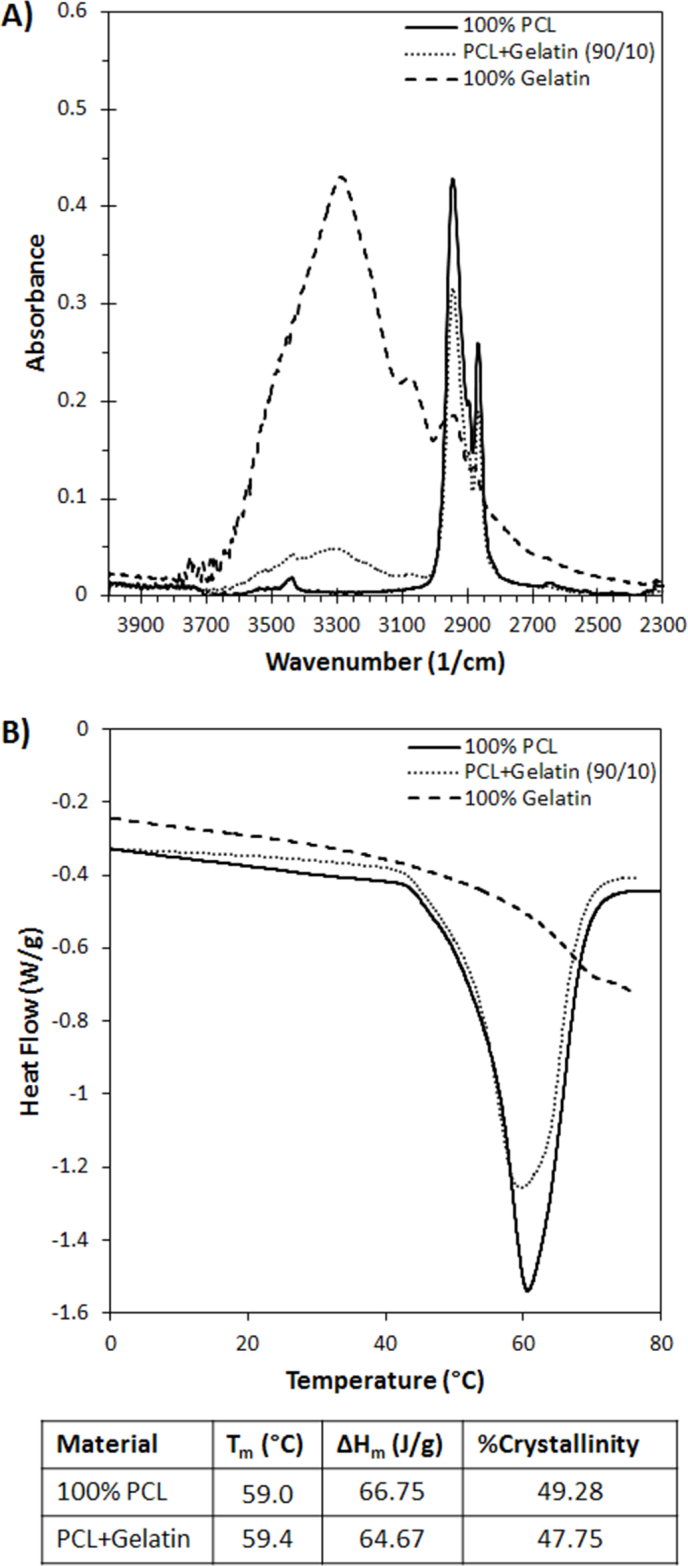
Polymer Characterization. A) Fourier Transform Infrared Spectroscopy (FTIR) of samples of electrospun gelatin, PCL, and PCL+Gel blend. The data PCL+Gel shows a characteristic N-H stretch peak for amines at 3300 1/cm. This confirms the presence of gelatin within the electrospun matrix. B) Differential Scanning Calorimetry (DSC) of samples of electrospun gelatin, PCL, and PCL+Gel blend. The data indicates that the addition of gelatin to the PCL does not drastically change the crystallinity or melting point of the PCL.

FTIR and DSC were performed to characterize the material properties of the synthesized electrospun nanomatrices. FTIR data (Fig 2A) shows a characteristic peak for amines at 3300 1/cm for the 100% gelatin sample, and no peak for the 100% PCL sample. For the PCL+Gel sample, a smaller peak is present indicating the presence of gelatin within the sample, the height of the smaller peak is 11% of the 100% gelatin peak, which confirms the ratio of PCL to gelatin in the PCL+Gel sample. To characterize the thermal properties of the material, DSC was performed. The DSC data (Fig 2B) indicates that pure PCL melts at 59°C and has a crystallinity of 49%, which is similar to other reported crystallinity values for electrospun PCL [7]. The addition of gelatin to the PCL did not alter the melting point, and only slightly decreased the enthalpy of fusion, indicating that the crystalline structure of the PCL fibers was not substantially altered by the addition of the gelatin.

**Fig 3:**
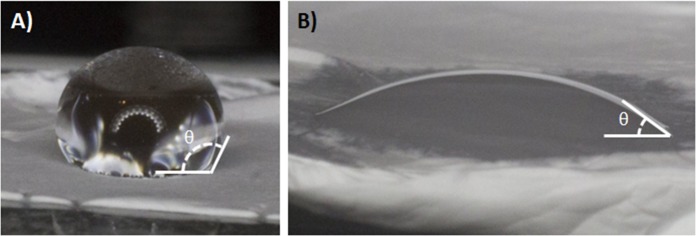
Hydrophilicity Quantification. A) A water drop on 100% PCL, the hydrophobic surface produces a large contact angle. B) A water drop on PCL+Gel surface, the gelatin increases the hydrophilicity of the surface producing a smaller contact angle. The data shows a clear change in contact angle induced by the addition of gelatin into the electrospun fiber mat.

The adsorption of biomolecules to the surface biomaterial is strongly linked to the hydrophilicity of the surface. To quantify the surface hydrophilicity of the electrospun materials, the contact angle was measured as shown in Fig 3. The contact angle for water on 100% PCL was found to be 124.7±8.2°, while the contact angle for water on PCL+Gel was found to be 25.7±6.4°.

### Cell adhesion response on fibers

**Fig 4:**
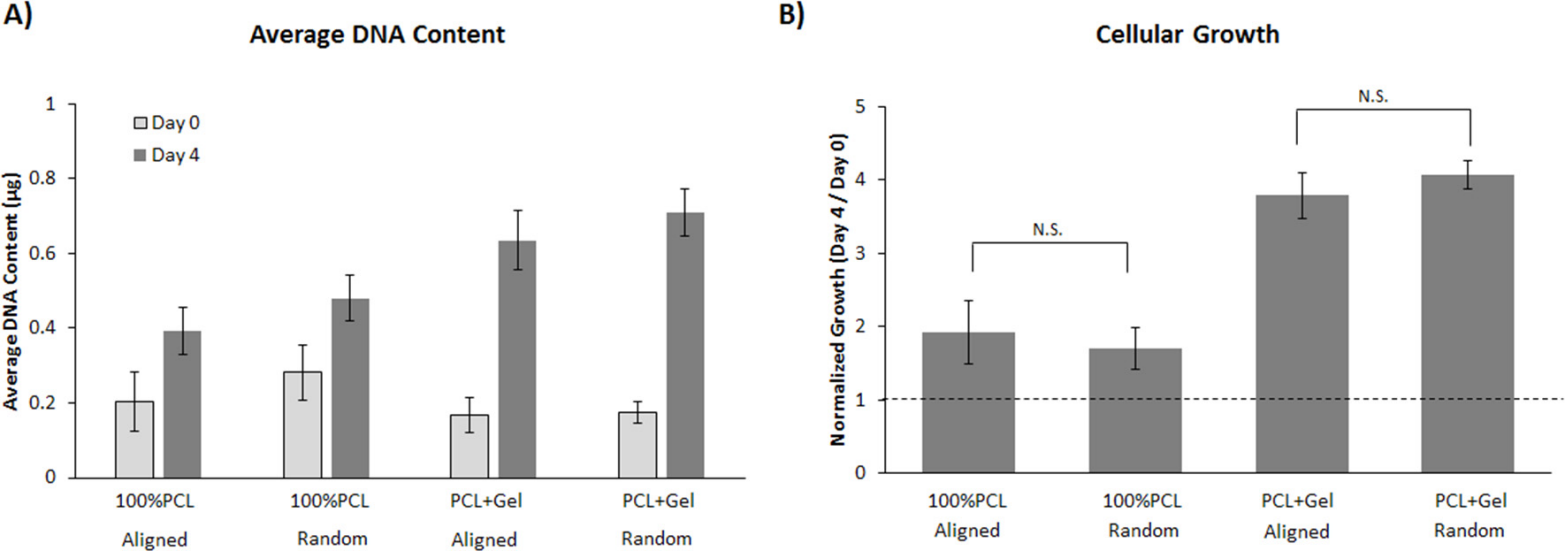
Proliferation assay of NIH 3T3 cells growing on various substrates. A) The average amount of DNA for cells grown on each substrate at 0 and 4 days. The DNA content is directly proportional to the number of cells. B) The cellular growth rate on various substrates as determined by the ratio of DNA content on days 0 and 4. This data indicates that orientation of the fibrous substrate does not influence the initial attachment or growth rate of NIH3T3 cells. However, the addition of gelatin does a substantially increase the growth rate over 100% PCL matrices (P<0.05, 2-way ANOVA, Tukey post-hoc). Error bars are ±SD.

NIH3T3 fibroblast proliferation and growth on electrospun matrices were quantified at 0 and 4 days (Fig 4). After cells were seeded on random or aligned PCL or PCL+Gel, they were allowed to adhere for 4 hours then washed and the remaining cells were quantified. The data in Fig 4 shows that on day 0, there is very little difference in cellular adhesion on 100% PCL compared to PCL+Gel. After 4 days, there was a statistically significant difference in the growth rate of fibroblasts on the scaffolds containing gelatin. At both 0 and 4 days there was no significant difference between groups of random and aligned fibers.

### Cell Alignment changes on fibers

**Fig 5:**
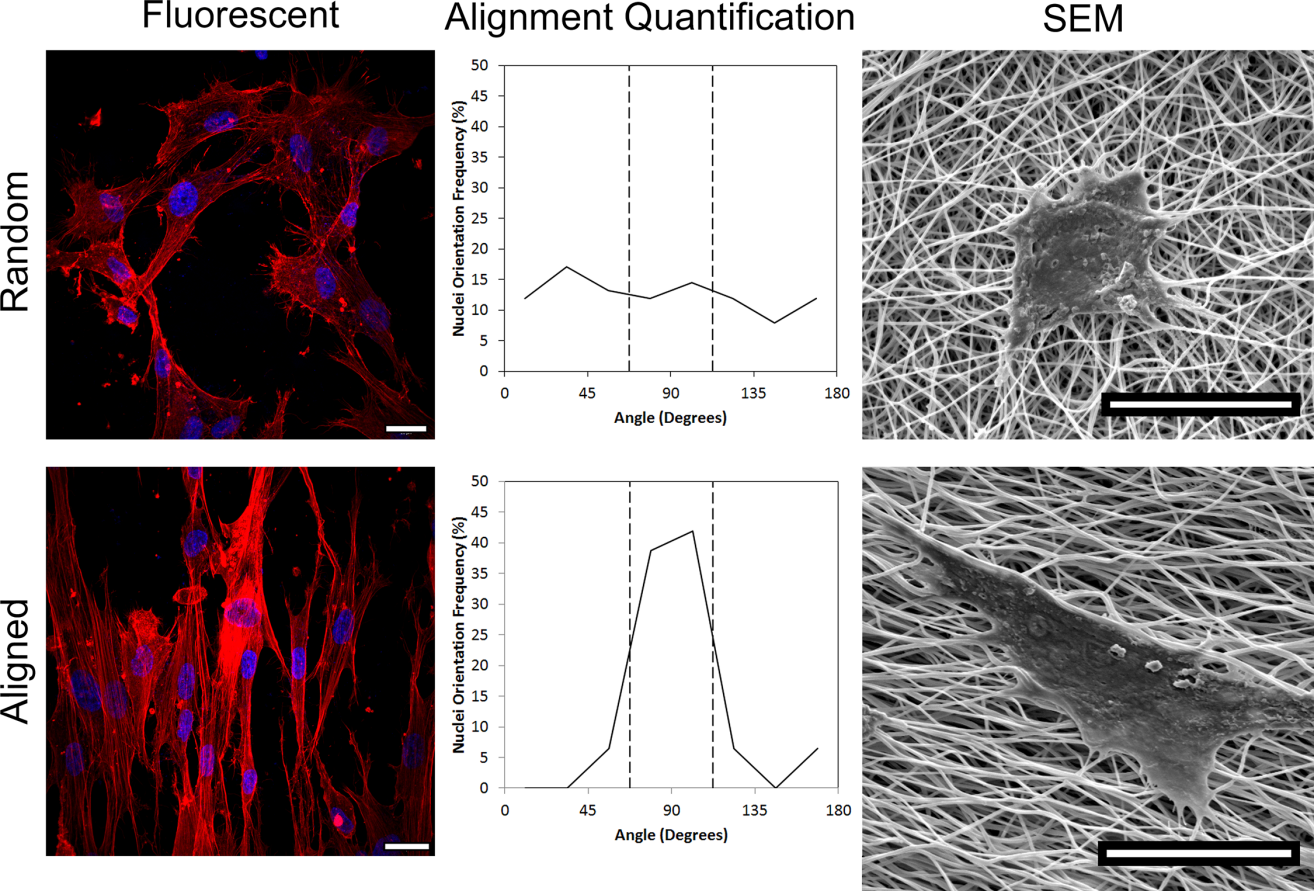
Microscope images showing cells growing on random (top) or aligned (bottom) electrospun PCL+Gel fibers. Fluorescent images are shown on the left with the cells stained to show the nuclei and stress fibers. The central column shows the distribution of cell orientations, confirming the cells on aligned fibers have a preferred orientation. Electron microscope images are shown on the right to illustrate the cellular interactions with the substrate. All image scale bars are 20μm.

To observe the morphological response of fibroblasts on PCL+Gel to substrate topology, adherent cells were imaged using fluorescent and electron microscopy. Fibroblasts growing on an aligned substrate showed a clear preference to elongate and orient themselves parallel to the direction of fiber alignment, while fibroblasts on randomly oriented fibers show no preferential orientation (Fig 5). When the overall orientation of cells was quantified, 64% of the nuclei on aligned substrates were aligned within +/- 22.5° of the mean direction compared to only 30% of cells on randomly oriented substrates (p = 0.021).

### Cell gene expression response on fibers

**Fig 6:**
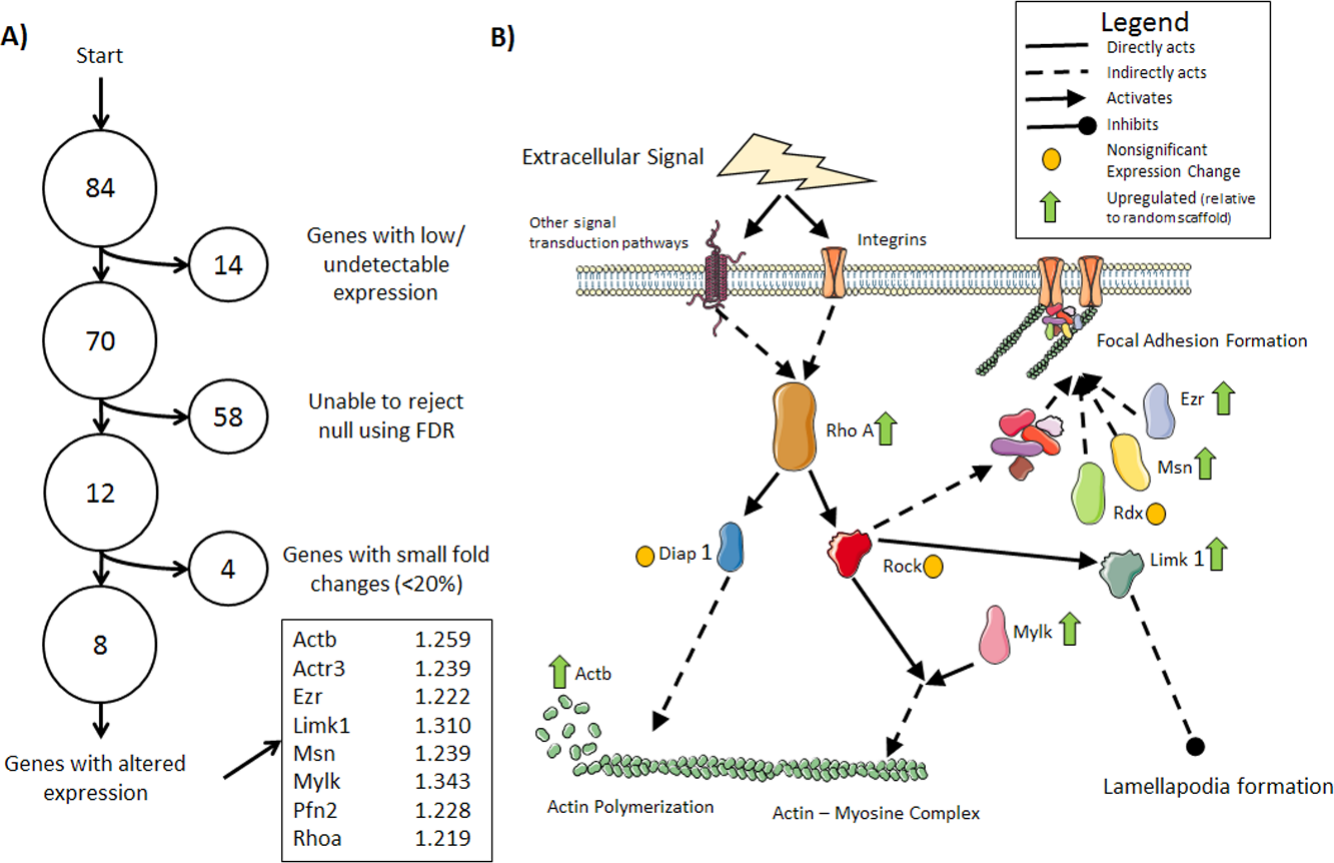
Gene Expression Microarray Results. A) A diagram showing the process for identifying genes with altered expression: the false discovery rate (FDR) was calculated using BH method to limit the FDR to at most 2 expected false positive results in the all rejected null hypotheses (FDR = 16.6%). These results were further limited to genes with the largest fold changes (fold change > 20%). The identified genes are listed along with their fold changes relative to randomly oriented scaffolds. B) Pathway diagram illustrating where the protein products of several of the identified genes work to promote actin production and polymerization and focal adhesion assembly.

To examine how fibroblasts respond to the topological signals of their substrate, a panel of cytoskeletal regulators was probed in a gene expression microarray. Of the 84 genes examined, the expression of 12 were found to be statistically different between the random and aligned scaffolds. Because statistical significance does not always equate to biological significance in gene expression studies, the genes identified as statistically significant were further limited by the magnitude of the fold change, resulting in 8 genes with altered expression (Fig 6A). Combining a fold-change magnitude threshold with a statistical significance threshold has been previously utilized to identify differentially expressed genes [24], [25]. The protein products of the upregulated genes were associated with actin polymerization and focal adhesion formation (Fig 6B).

## Discussion

An important goal in tissue engineering is the ability to produce a platform which will induce a desired cellular phenotype to replicate that which is observed in the native tissue. One key component of this goal is spatial organization of adherent cells that recapitulates the natural tissue architecture. Electrospun scaffolds are frequently used as a nanoscale biomaterial to provide control over cellular phenotype and behavior through topological and biochemical mechanisms. Here, we have more closely examined how the microtopological properties of electrospun substrates influence the behavior of adherent fibroblasts.

Though often used in electrospun matrices because of its desirable mechanical properties, PCL is a relatively hydrophobic material. This hydrophobicity can lead to impaired cellular adhesion due to the lack of hydrophilic protein adsorption. To remedy this, we fabricated an electrospun scaffold composed of PCL blended with gelatin in a 90/10 ratio. The composition of the resulting scaffold was confirmed using FTIR and DSC. When the hydrophobicity was measured, it was found that the contact angle for the PCL alone was nearly five times larger than the contact angle of PCL+Gel. Typically, the threshold for defining a hydrophobic material is a contact angle of 90° or more, while a hydrophilic material has a contact angle smaller than 90°. The measured contact angle for 100%PCL was about 125° while the addition of gelatin reduced the angle to only 25°.

The addition of gelatin to the electrospun PCL also had a profound impact on cellular growth. While there seems to be no substantial difference in cellular attachment during the first several hours after seeding, a statistically significant difference in growth rate is evident after 4 days of culture. The initial similarity in cell adhesion suggests that the hydrophobicity of 100%PCL does not initially depress cell seeding efficiency, but does negatively influence cell proliferation. It is known that gelatin contains functional peptide sequences associated with integrin binding. The most well-known of these is the RGD sequence of arginine-glycine-aspartic acid, though it has been shown that others exist as well as the ability to bind to sites on fibronectin [26], [27]. It is of interest to note that the orientation of the fibers within the matrix did not have a substantial influence on adhesion or proliferation in either PCL+Gel or PCL alone.

Our experiments have shown that even with similar growth rates, there is a marked difference in cellular behavior when cultured on aligned vs randomly oriented electrospun fibers. The influence of nanoscale fiber orientation on cellular morphology has been described for a variety of cell types, including astrocytes, mesenchymal stem cells, osteoblasts, and smooth muscle cells to name a few. The mechanisms by which this occurs is not fully understood. It has been previously shown that cells on a surface that has been pre-stressed in one direction to yield a smooth anisotropic substrate will elongate preferentially in the direction of highest substrate stiffness [28]. This mechanotransduction explanation for cell reorientation on aligned nanofibers is possible as aligned nanofibers are known to produce anisotropic mechanical properties within the scaffold [29]. It is also possible that topology alone exerts some influence on cell morphology as cells grown on microgrooved substrates with nearly isotropic mechanical properties also show a preferential orientation [30].

While some genes have been identified as up or down regulated due to electrospun fiber alignment with the substrate in various cell types, the authors are not aware of any such studies using fibroblasts. To identify some of the genes involved in substrate induced cell morphology changes, a microarray study of 84 cytoskeleton regulators was performed. The microarray data indicated 8 genes that were statistically different between random and aligned substrates and had an expression difference above a given threshold. The protein products of these 8 upregulated genes have been previously linked to actin polymerization, focal adhesion formation, actin production, and mechanosensitivity. The full list of genes examined and the microarray results are provided in S1 Table.

One of the genes identified as upregulated on aligned fibers is *Rhoa*. While the gene encoding its kinase, *Rock*, was not found to be statistically different between the groups, the RHOA-ROCK pathway has been identified as an important mechanotransduction pathway in numerous studies [31]. The downstream effects of the *Rhoa* protein product includes actin polymerization, stress-fiber formation, focal adhesion assembly, and actin-myosin complex contraction [32], [33]. In addition to *Rhoa*, we identified *Ezr* and *Msn* to have increased expression in cells grown on aligned fibers. The products of these genes are known to be components of focal adhesions. Our results indicates an increased in expression of genes whose products promote production and polymerization of actin in fibroblasts as well as an increased production of focal adhesion components on aligned nanofibrous scaffolds compared to randomly oriented nanofibers.

The closest comparable published result is a microarray analysis of fibroblasts on microgrooved quartz substrate [34]. The authors of this study did not discuss cytoskeletal regulation changes as they were focused on nuclear reorganization as an explanation for gene expression alterations induced by topographical cues. Additionally, the use of a deformable substrate in our study combines the topological cues with mechanical anisotropy to better recapitulate the environment of both native and engineered tissues.

As with any study, this work is subject to certain limitations. One technical challenge common to all microarray experiments is the multiple comparisons problem. While an uncorrected t-test would seem to suggest a highly statistically significant result, a large number of genes tested increases the likelihood of a type I error. Several approaches have been suggested to correct for multiple comparisons without sacrificing statistical power; the Benjamini–Hochberg (BH) method is well established for increasing the number of null hypothesis rejections at the expense of less stringent control over type I errors. We attempted to mitigate the possibility of false discoveries by applying a fold-change threshold to the genes identified as statistically significant using the BH method. While there is no guarantee that the genes with smaller expression differences are associated with false discoveries, there is a larger confidence in the biological significance of larger changes in gene expression.

## Conclusions

In the present study, we examined the influence of substrate nanofiber orientation on the expression of cytoskeleton regulators in fibroblasts. The nanofibrous substrates used were composed of electrospun PCL+Gel, the composition of which was confirmed by FTIR. It was shown that we have fabricated random and aligned electrospun PCL+Gel scaffolds and shown them to be suitable for cell culture with an increased cell growth rate over scaffolds made from PCL alone. Fibroblasts grown on the matrix of aligned nanofibers altered their morphology to elongate in a preferred orientation parallel to the underlying fibers. Gene expression analysis found that fibroblasts on aligned matrices upregulated genes associated with actin production, actin polymerization, and focal adhesion formation. This is the first time that electrospun substrate modulated gene expression has been shown with fibroblasts, and these results deepen the understanding of the mechanism by which fibroblasts interact with nanofibrous substrates.

## Supporting Information

S1 Table Full list of genes included in the expression microarray. The listed fold change and results are for the cells on the aligned scaffold relative to cells on the random scaffold.
(CSV)
